# Using Microalgae as a Sustainable Feed Resource to Enhance Quality and Nutritional Value of Pork and Poultry Meat

**DOI:** 10.3390/foods10122933

**Published:** 2021-11-28

**Authors:** Cátia F. Martins, David M. Ribeiro, Mónica Costa, Diogo Coelho, Cristina M. Alfaia, Madalena Lordelo, André M. Almeida, João P. B. Freire, José A. M. Prates

**Affiliations:** 1 CIISA—Centre for Interdisciplinary Research in Animal Health, Faculdade de Medicina Veterinária, Universidade de Lisboa, 1300-477 Lisbon, Portugal; catiamartins@isa.ulisboa.pt (C.F.M.); monicacosta@fmv.ulisboa.pt (M.C.); diogocoelho@fmv.ulisboa.pt (D.C.); cpmateus@fmv.ulisboa.pt (C.M.A.); 2 LEAF—Linking Landscape, Environment, Agriculture and Food, Instituto Superior de Agronomia, Universidade de Lisboa, 1349-017 Lisbon, Portugal; davidribeiro@isa.ulisboa.pt (D.M.R.); mlordelo@isa.ulisboa.pt (M.L.); aalmeida@isa.ulisboa.pt (A.M.A.); jpfreire@isa.ulisboa.pt (J.P.B.F.)

**Keywords:** microalgae, pork, meat, poultry, sustainability, nutritional quality

## Abstract

Cereal grains and soybean meal are the main feedstuffs used in swine and poultry feeding, two of the most consumed meats and of key relevance to food security worldwide. Such crops are grown mostly in North and South America and transported over large distances creating sustainability concerns and, furthermore, are in direct competition with human nutrition. Alternatives to these ingredients are, thus, a pressing need to ensure the sustainability of swine and poultry production. Microalgae seem to be a viable alternative due to their interesting nutritional composition. The use of different microalgae in monogastric feeding has been addressed by different researchers over the last decade, particularly their use as a supplement, whilst their use as a feed ingredient has been comparatively less studied. In addition, the high production costs of microalgae are a barrier and prevent higher dietary inclusion. Studies on the effect of microalgae on meat quality refer mostly to fatty acid composition, using these either as a functional ingredient or as a feedstuff. Within such a context and in line with such a rationale, in this review we address the current research on the topic of the use of microalgae in poultry and swine nutrition, particularly aspects concerning pork and poultry meat quality and nutritional traits.

## 1. Introduction

Projections indicate that global population will double by 2050, especially the urban population, with a growing income. Consequently, cereal and meat production need to increase to meet the foreseeable increased demand for foodstuffs. Corn and soybean meal are the basis of compound feeds for those monogastric animals, which are in most demand by the consumer [1]. The lack of sustainability of such crops due to droughts, drastic climate changes and competition with human nutrition are the main reasons for the need to find alternatives to these feedstuffs. Ideally, the novel feed resources should have high nutritional value, be able to optimize the use of land and water and assure animal product quality in a sustainable system [2].

Microalgae are a promising source of protein for both food and feed. They do not require arable land and are produced in photobioreactors or race-way ponds using, for instance, saltwater or wastewater. Additionally, one of the advantages associated with microalgae production is the possibility of using them as bio-sequesters of carbon dioxide, which offers a promising scenario for the reduction of greenhouse gas emissions by, for example, integrating microalgal production into biorefineries [3]. The nutritional profile of microalgae, amongst other factors, varies considerably according to the algae species. In general, microalgae are characterized by protein, carbohydrate and lipid contents that are, at least, comparable to conventional feedstuffs. In this context, the use of microalgae in animal feed has been an object of study as nutrient supply but also as a source of bioactive compounds that can improve animal immune response, disease resistance, antiviral and antibacterial action and gut function and stimulate probiotic colonization [4]. Microalgae are furthermore a relevant source of pigments, vitamins, minerals and fatty acids (FA), particularly eicosapentaenoic (EPA) and docosahexaenoic (DHA) acids, which are known to improve meat quality of pigs and broilers [4]. Similarly, other relevant marine resources like macroalgae have also been studied by the scientific community as alternative ingredients, emphasizing the effects on growth performance and meat quality of livestock animal species [5].

In line with all of the above, this work reviews the currently available literature regarding the effects of dietary microalgae on quality traits and nutritional value of pork and poultry meat.

## 2. Microalgae: Definition and Properties

The broad spectrum of microalgae includes more than 100,000 species divided into four distinct groups: eukaryotic diatoms (*Bacillariophyceae*), green algae (*Chlorophyceae*), golden algae (*Chrysophyceae),* and blue-green algae (*Cyanophyceae*) [4]. They are mostly autotrophic, since carbon dioxide is the carbon source and sunlight the energy source, but there are also heterotrophic microalgae that use organic carbon instead of sunlight as energy source. The latter are easily cultivated in bioreactors and easily used for biomass production. Microalgae can grow in non-agricultural lands such as coastal lands, desert or semiarid areas. Their cultivation requires freshwater, saltwater or wastewater from agricultural, domestic, or industrial origins [3].

Microalgae have an interesting composition of proteins, carbohydrates, lipids, vitamins, minerals and bioactive compounds, such as carotenoids. This composition of macro- and micronutrients depends on several factors, like species, strain, growth conditions and biomass status (whole or defatted algae meal) [6]. Spirulina (*Arthrospira* sp.), *Chlorella* sp. and *Schizochytrium* sp. have been the most widely used microalgae in animal production.

Spirulina is known as a source of protein, which varies between 60 and 70% of dry weight. It is also a rich source of antioxidants, such as β-carotene and vitamin E, and has a high content of FA, mainly γ-linolenic acid (GLA) [7]. The latter can lead to an increase in the level of this FA in the meat [8]. The range of variation for crude carbohydrates, crude fat and ash contents are on a dry weight basis: 17.8–22.6%, 1.8–7.3% and 6.5–9.5%, respectively [4].

*Chlorella* sp. has a protein concentration in the range of 50–60% of dry weight and is considered an important source of cobalamin (vitamin B_12_) [4,9]. Comparatively to Spirulina, it has similar contents of carbohydrates and ash and a higher level of crude fat (12.6 points more, on average) and of *n*-3 PUFA (9 points more, on average) [4].

*Schizochytrium* sp. is of particular interest for its oils, which are particularly rich in DHA. Several studies used it as a feed supplement under the form of DHA-Gold extract [6]. With a lower protein content than the other microalgae (about 12.1% of dry weight), it is known by its higher crude fat content (38.0 to 71.1% of dry weight).

The nutritive value of microalgae for livestock production depends on algal species and proximate composition, as well as the adaption of animals to the feedstuff [4]. Microalgae have been successfully used in feeding trials on ruminants, rabbits, broilers and pigs [10,11,12,13]. However, several authors have reported issues with the recalcitrant cell walls of microalgae that grant resistance to predation and desiccation. This recalcitrance is the result of the presence of a diverse and complex matrix of cross-linked insoluble carbohydrates in the cell wall [14]. Thus, the microalgal cell wall is largely indigestible by monogastric animals and, so, it is necessary to develop novel technologies to improve microalgal nutrient utilization and ease the cost-effective use of microalgae for the animal feed industry [15,16]. Carbohydrate-Active enZymes (CAZymes) have been studied to enhance the digestion of dietary complex carbohydrates from microalgae [17,18] and applied in pig and poultry feeding trials to prove their effectiveness [19,20,21,22,23].

In parallel, there is the issue of the inefficient available technology for microalgae production that strongly limits their use. Indeed, the production of microalgae is, in general, very expensive. However, such costs will likely be reduced in the coming years due to optimization of production technologyand increasing microalgae productivity [24]. These authors referred, for instance, to the differentiation of final price of biomass related to cultivation strategies and water supply. For example, small-scale for specialized applications has a higher price (more than 4€ per kg of microalgae) than wastewater cultivation combined with carbon dioxide capture from industrial flue gases [24].

Indeed, microalgae after production are dried resulting in a powder that can be immediately incorporated into animal diets. This procedure extends the shelf-life of the product and facilitates the transportation and storage of microalgae products. Such methods (freeze drying, spray drying, etc.) are, however, extremely costly and raise sustainability issues due to the use of energy, obtained mostly from fossil fuels.

## 3. Meat Quality and Nutritional Value

The global consumption of meat is projected to increase by 14% in 2030, mainly poultry meat and pork (OECD, 2021). Pork is one of the most commonly consumed meats worldwide, and the most commonly consumed in Europe. Thus, it is an important source of protein and fat for human diet. However, the lipid nutritional value of this meat is poor due to the low levels of the beneficial omega-3 (*n-*3) long-chain polyunsaturated fatty acids (*n*-3 LC-PUFA), EPA and DHA [25]. Therefore, during recent years, the scientific community has been trying to improve the sensory qualities and nutritional value of pork by controlling its FA profile [26]. Poultry meat, also widely consumed worldwide, provides high-quality protein and low amounts of fat, which is beneficial to human nutrition and health [5].

The health benefits of increasing intake of *n*-3 FA are mainly associated with a reduced risk of cardiovascular disease and an improvement in cognitive functions in childhood and older age. To meet the recommended daily requirements of EPA and DHA in humans, it is necessary to consume products rich in these beneficial FAs [27]. Algae have been used in animal feed to increase the *n*-3 FA content of animal products [28]. Tissues from monogastric animals, such as pigs and poultry, are susceptible to FA changes through dietary modification and this is a viable strategy to increase *n*-3 FA in their products. However, the addition of *n*-3 LC-PUFA can lead to adverse effects, particularly by increasing the lipid peroxidation of meat products. Lipid peroxidation decreases the nutritive value of meat and, also, generates oxidation products (malondialdehyde and volatile compounds) causing off-flavor, off-tasting and color changes [29]. To control these problems, some authors advocate limiting PUFA content in swine and poultry diets and/or associating it with antioxidants, such as vitamin E and selenium [30].

As already mentioned, microalgae contain high amounts of *n*-3 LC-PUFA and thus, represent an unexploited natural resource with well-known beneficial health effects for both humans and animals [31]. In particular, the increase of *n*-3 PUFA content in meat and meat products is the most referenced parameter in several studies on the subject [8,19,20,21,22,28,32,33,34,35,36,37,38,39,40,41,42,43,44,45]. Furthermore, due to the inefficiency of lipid extraction process during biofuel production, the residual fiber obtained as a by-product has a high content of *n*-3 LC-PUFA and, thus, could be a valuable sustainable feed source [46].

Meat quality is evaluated by classical methodologies but, in recent years, it has been associated with innovative methods, such as omics, which allow the analysis of tissue metabolism at the molecular level in order to understand meat quality traits [47].

## 4. Production of Pork with Dietary Microalgae

The literature related to the effects of different microalgae on quality traits and nutritional value of pork is summarized in Table 1.

The inclusion of Spirulina, as a supplement, was studied by Simkus et al. [13], who used daily 2 g of fresh microalga biomass plus forage for each pig weighing from 30.6 to 96.4 kg. The ADG was 9.26% higher in microalgae-fed pigs than in the control group with no effect on backfat thickness, but the amount of intramuscular fat in meat decreased by 0.33% [13]. The authors did not examine the FA composition and sensory properties, which, afterwards, were evaluated by Altmann et al. [8]. According to these authors, the dietary inclusion of the same microalga at high levels (6.60–12.5%) leads to stronger overall odors and a more astringent aftertaste in meat compared to the control group. This could be attributed to a different FA composition with higher PUFA contents, mainly C18:3*n*-3 and C18:3*n*-6 in the subcutaneous fat, with no effect on lipid peroxidation. Recently, Martins et al. [19] fed weaned piglets for 4 weeks with 10% Spirulina diets supplemented or not with lysozyme and found that the meat quality traits were not negatively affected by the addition of this microalga. Specifically, the authors reported higher tenderness and flavor scores, as well as an increase in C18:3*n*-6 and total carotenoids content in the meat of Spirulina groups. Overall, the above studies had in common increased PUFA levels. This is not surprising because Spirulina is known to have a high PUFA content, particularly of C18:3*n*-6. Moreover, the high PUFA content was not associated with increased thiobarbituric acid reactive substance (TBARS) values [8].

The same research group, through reports by Martins et al. and Coelho et al. [20,21], studied the effects of 5% of *Chlorella vulgaris* in the diets of post-weaning and growing pigs, respectively. Both studies demonstrated an improvement in the nutritional value of pork, through an increase in total carotenoids and *n*-3 PUFA content. Furthermore, they simultaneously demonstrated that the use of carbohydrases had a minor impact on meat quality, with no influence in growth performance. Seemingly, at this level of incorporation, there is no need for the use of enzymes to improve pig’s digestive function.

Sardi et al. [32] reported that moderate levels of *Schizochytrium* sp. (0.50%) in barrows’ diets increased DHA content of loin and backfat. Although the supplementation with 0.25% for 4 or 8 weeks caused similar levels of DHA enrichment (50 and 40 mg DHA/100 g of *longissimus lumborum* (LL), respectively) compared to the control group, 0.5% of microalga for 8 weeks led to higher content of DHA (70 mg/100 g LL). The treatments did not affect animal performance, meat pH values, meat color or iodine amount in subcutaneous fat and EPA content in LL and backfat. In parallel, Vossen et al. [33] investigated the effects of dietary supplementation with 0.3, 0.6 and 1.2% of *Schizochytrium* sp. for 45 days in finishing pigs from 75 to 110 kg and observed an increase of DHA content in pork products (loin and dry cured ham). Nonetheless, lipid peroxidation increased, as a consequence of DHA enrichment, in processed products, which was not found in fresh meat. Previously, Meadus et al. [34] using the same microalga and similar incorporation levels (0.06, 0.6 and 1.6%) in pigs reported a linear increase of DHA content in bacon. In this study, off-odor and off-flavors were detected by a trained sensory panel in bacon from pigs fed the highest inclusion level, which were associated with higher lipid peroxidation. The same authors performed a study with the direct injection of DHA into pork loins and established an increase of 146 mg DHA/100 g serving meat without any undesirable taste being detected by the trained sensory panel [48].

Moran et al. [28,38] using *Schizochytrium* sp. (*Aurantiochytrium limacinum*) also performed two different studies. Firstly, they used lower levels of dietary incorporation (0.25 and 0.50%) for pigs with an initial weight of 27.9 kg for 114 days and found an enrichment of DHA in LL and backfat. Subsequently, the authors studied either the effect of reducing the feeding period (last month before slaughter) or a higher inclusion level (1%) of microalga. Significant changes in FA profiles of pork LL and backfat were observed, pointing out the increase of DHA content in the microalgae-fed group. Thus, they found similar increases in DHA content in LL and backfat over long and short feeding periods and these findings demonstrated that this microalga effectively increased the *n*-3 PUFA content of pork.

De Tonnac et al. [35], studied the FA composition of different tissues (*longissimus thoracis*, LL, *semimembranosus* and diaphragm) enriched with dietary *n*-3 PUFA from *Schizochytrium* sp. (0.9, 1.9 and 3.7%) in finisher pigs (from 50.7 to 115 kg). They found that DHA deposition depends on tissue location, where adipose tissues located in extremities revealed higher *n*-3 and *n*-6 PUFA than tissues in the middle of the carcass. Specifically, the percentages of C20:4*n-*6, C20:5*n-*3 and C22:6*n-*3 increased in tissues from pigs fed with microalga. However, this high PUFA content in microalgae-fed pigs increased pork’s lipid peroxidation and fish odor, discernible by a trained sensory panel [36]. The authors concluded that a limit below 1.5% of microalga incorporation should be used in swine feed to avoid negative effects on the oxidation susceptibility and sensory parameters of pork. In a previous study by the same authors, using finisher pigs (from 64.6 to 115 kg) and 0.94, 1.85, 2.74 and 3.61% of microalga demonstrated that increasing DHA intake down-regulates the activities and gene expressions of key lipogenic enzymes involved in FA metabolism, mainly in the liver [37]. Kalbe et al. [49] tested a higher inclusion level (5%) of *Schizochytrium* sp. in diets of finisher pigs and highlighted the accumulation of DHA and EPA in *longissimus thoracis* and *semitendinosus* of microalgae-fed pigs without significant effects on meat quality traits. However, protein content increased in the *longissimus thoracis* muscle due to DHA-rich microalga supplementation, which can induce muscle protein synthesis [50].

Importantly, several authors have indicated that long-term algae supplementation with lower concentrations had similar effects on enrichment levels than short-term supplementation with higher concentrations. Concerning the pork´s fatty acid composition, there were no differences between the lowest level of supplementation over a longer period and the highest level of supplementation over a shorter period [28,32,33,38]. The most common studies in this area have focused on supplementation with microalga *Schizochythrum* sp., which has shown increasing levels of EPA and DHA in pork without negatively impacting swine productivity [32,33,34,35,36,37,49].

## 5. Production of Poultry Meat with Dietary Microalgae

In Table 2 the main effects of different microalgae used in poultry diets on quality traits and nutritional value of poultry meat are presented.

The microalga Spirulina is one of the most commonly studied in the field of broiler feeding trials. Venkataraman et al. [51] studied this microalga as an ingredient (12–17% of sun-dried Spirulina) in broiler diets and found no influence on growth and meat quality traits. However, Spirulina carotenoids were incorporated into tissues, where the skin, breast and thigh muscles and depot fat from fed-microalga broilers showed deeper color pigmentation than the control group. Similarly, Pestana et al. [22] demonstrated that breast and thigh meats from broilers fed 15% Spirulina had higher values of yellowness and total carotenoids. The latter authors also found an increase in SFA and a decrease of *n*-3 PUFA and α-tocopherol when compared to the control group [22].

Additionally, Toyomizu et al. [52] studied the incorporation of 4% and 8% of Spirulina in male broilers for 16 days and found differences in the values of yellowness and redness of their meat. The authors suggested that dietary Spirulina could be an interesting way to control broiler meat color, as it did not reach the extremes of dark or light meat, which are not recognized as optimal by consumers. Altmann et al. [53] verified improvements in meat quality, such as increased water-holding capacity and decreased off-flavors, despite the dark reddish-yellowish meat color obtained for 9.7–11.8% of Spirulina in broiler diets.

At lower levels of incorporation, Park et al. [54] tested 0.25, 0.5, 0.75 and 1% Spirulina in broilers’ diets and detected decreased loss of breast meat after 7 days of storage compared to the control group. Along the same lines, Bonos et al. [39] explored the addition of 0.5 and 1% of Spirulina to basal diets of broilers for 42 days and, although without effects on lipid peroxidation, PUFA levels were increased in breast and thigh meat, specifically DHA in the thigh meat.

Alfaia et al. [23], using 10% *C. vulgaris* in broiler diets, detected a slight improvement in meat quality and lipid nutritional value with increased total carotenoids, yellowness and tenderness in breast and thigh meats. These findings were also detected by the aforementioned authors through a higher incorporation level of other microalgae [22]. For the first time, Oh et al. [55] studied fermented *C. vulgaris*, as a supplement (0.1 and 0.2%) in duck diets, and found improvements in duck meat: increased breast meat yellowness and leg meat lightness and yellowness, as well as increased shear force, pH and water-hold capacity in breast meat but not in leg meat.

Several authors focused their work on *Schizochytrium* sp., highlighting the positive increment of DHA proportion in meat [4,40,41,42,44,45,56]. Mooney et al. [41] incorporated this microalga (2.8 and 5.5%) in broiler feeds that yielded an *n-*3 PUFA enriched broiler breast product. They considered that 2.8% microalga was the best level without compromising flavors or lipid quality, because 5.5% was not acceptable by consumer panelists due to the significantly reduced flavor compared to the control meats. Khan et al. [56] demonstrated the effect of DHA-rich microalga (2% of *Schizochytrium* sp.) along with methionine supplementation enriching meat with *n*-3 FA, associating this amino acid supplement with meat tenderness and color. Baeza et al. [42] reported that microalgae supplementation (0.5 and 2% *Schizochytrium* sp.) increased long chain *n*-3 FA and susceptibility to oxidation in breast meat and the presence of off-flavors in thigh meat of broilers. The authors recommended limiting the level of incorporation in broiler feed and the association of algae with linseeds to preserve high contents of *n*-3 LC-PUFA and linolenic acid in breast meat. This allows avoiding the negative impact of microalga on the oxidation susceptibility and the sensory parameters of breast and thigh meat, respectively.

Long et al. [57], focusing on supplementation diets with 1 or 2% of *Schizochytrium limacinum* to replace soybean oil in diets of broilers from 1 to 42 days-old, found higher levels of EPA and DHA in breast and thigh meat compared to the control group. Moran et al. [43] studied broilers receiving 0.5, 2.5 and 5% supplementation of *Schizochytrium* sp. (*Aurantiochytrium limacinum*). Significant increases in meat DHA content were observed, thus increasing the nutritional value of meat. However, at a higher level (7.4%) of dietary *Schizochytrium* sp. [44], the overall acceptability of meat was negatively affected. In turn, 3.7% proved to be efficient in enhancing LC-PUFA in meat without affecting its sensory properties. The same authors studied complementary supplementation with 7.4% of *Schizochytrium* sp. when reducing dietary crude protein from 21% to 17% in broiler diets. They reported the promotion of carcass yield and meat FA profile, but meat overall acceptability and oxidative stability were reduced [45]. To control these changes in taste and lipid peroxidation, one should consider incorporating this microalga at a lower inclusion rate, as studied by Yan and Kim [40]. They found that 0.1 and 0.2% of *Schizochytrium* sp. enhanced the FA composition of breast meat.

In line with the microalga *Schizochytrium* sp. research, *Crypthecodinium cohnii* has been studied as potential substitute to fisheries-derived oils. The results obtained show the potential of these microalgae as DHA sources, but also indicate the need for an EPA source to fully replace fish oil [58]. Schiavone et al. [59] studied 0.5% of *Crypthecodinium cohnii* supplementation in diets of Muscovy ducks during the last 3 weeks of life. The authors found a significant increase in DHA content in duck’s breast meat, without influencing color, pH, oxidative stability and sensory characteristics. There are also several studies that used defatted green microalgae biomass resulting from biofuel production to create poultry products enriched with *n*-3 FA [60,61]. Gatrell et al. [60] used 2, 4, 8 or 16% of defatted *Nannochloropsis oceanica* in broiler diets and found a linear increase in *n*-3 FA, EPA, DHA in breast and thigh tissue. Tao et al. [61] studied 10% of defatted *Nannochloropsis oceanica* and found similar results with enrichment of EPA and DHA in breast and thigh meat of broilers.

## 6. Conclusions and Challenges

The use of microalgae in swine and poultry feeding has been shown to improve meat quality. Lower levels of microalgae supplementation seem to have some advantages in meat quality without negatively impacting animal growth. However, there is an inconsistent relationship between higher levels of supplementation and meat quality traits. Overall, microalgae may be used as natural ingredients or supplements in animal diets to meet the demand for novel feedstuffs. In addition, microalgae are interesting dietary sources of protein and energy, as well as an alternative to synthetic additives in the feed. Nevertheless, it is important to stress that microalgae nutrient availability, as well as their production costs, have to be considered in order to optimize their use in the context of animal feeding, performance and quality of meat products.

## Figures and Tables

**Table 1:** Summary of main effects of dietary microalgae on pork quality traits and nutritional value.

Microalga	Inclusion Level	Animal-Initial Weight LW-Final Weight LW or Trial Duration	Main Findings	Reference
*Arthospira platensis*	0.200%	Grower pigs-30.6–96.4 kg	Microalga had no effect on protein, color, pH, cooking loss, tenderness and backfat thickness; decreased the amount of intramuscular fat in meat	[13]
*Arthrospira platensis*	8.3 and 12.5% (1st period—25–50 kg LW), 6.6 and 9.9% (2nd period—51–75 kg LW) and 9.5 % (3rd period—more than 75 kg LW)	Barrows-25–110 or 122 kg	Microalga influenced the FA composition of backfat with increased PUFA levels. Meat quality was not compromised	[8]
*Arthrospira platensis*	10.0%	Weaned piglets-12.0 kg-4 weeks	No significant effects on meat quality traits with the dietary microalga	[19]
*Chlorella vulgaris*	5.00%	Weaned piglets-11.2 kg-2 weeks	Microalga improved total carotenoids and *n-*3 PUFA content in meat	[20]
*Chlorella vulgaris*	5.00%	Grower pigs-59.1–101 kg	Microalga improved total carotenoids and *n*-3 PUFA content in meat	[21]
*Schizochytrium* sp.	0.250% (for 4 or 8 weeks) and 0.500% (for 4 weeks)	Barrows-118–160 kg	Microalga had no effect on backfat thickness; feeding 0.50% microalga over 4 weeks prior to slaughter increased the DHA content and decreased *n*-6/*n*-3 ratio	[32]
*Schizochytrium* sp.	0.300, 0.600 and 1.20%	Finisher pigs-75–110 kg	Microalga increased lipid peroxidation and EPA and DHA contents in dry-cured hams; no effect on proximate composition, color, pH and TBARS values	[33]
*Schizochytrium* sp.	0.06, 0.60 and 1.6%	Finisher pigs-80–110 kg	Microalga increased DHA content and lipid peroxidation of bacon. Over 0.6% inclusion, consumer acceptability was reduced due to the development of off-flavors during and after cooking bacon	[34]
*Schizochytrium* sp.	0.250 and 0.500%	Grower pigs-27.9 kg–17 weeks	Microalga increased DHA content of loin and backfat	[38]
*Schizochytrium* sp.	1.00%	Finisher pigs-117–140 kg	Microalga increased EPA, DHA and *n*-3/*n*-6 ratio in *longissimus lumborum muscle*	[28]
*Schizochytrium* sp.	0.900, 1.90 and 3.70%	Finisher pigs-50.7–115 kg	Microalga increased C20:4, C20:5 and C22:6*n*-3 contents in the tissues studied; DHA deposition depends on tissue location	[35,36]
*Schizochytrium* sp.	0.940, 1.85, 2.74 and 3.61%	Finisher pigs-64.6–115 kg	Increasing dietary DHA reduced the activity of lipogenic enzymes in the liver and inhibited the expressions of genes involved in FA metabolism	[37]
*Schizochytrium* sp.	7.00% (piglet diet)/5.00% (grower pig diet)	Grower pigs-9.46–104 kg	Microalga increased DHA in *longissimus thoracis* and *semitendinosus* muscles	[49]

**Table 2:** Summary of main effects of dietary microalgae on poultry meat quality traits and nutritional value.

Microalga	Inclusion Level	Animal-Initial Age-Trial Duration	Main Findings	Reference
*Arthrospira platensis*	0.250, 0.500, 0.750 and 1.00%	Broilers-1-day old-5 weeks	Microalga decreased drip loss of breast meat after 7 days of storage	[54]
*Arthrospira platensis*	0.500 and 1.00%	Broilers-1-day old-6 weeks	Microalga had no effect on the lipid peroxidation of breast and thigh meat. Increased PUFA, EPA, DPA and DHA content in breast and thigh meat, more pronounced in the latter	[39]
*Arthrospira platensis*	4.00 and 8.00%	Male broilers chicks-21 days old-2 weeks	Increased the pigmentation of meat (yellowness and redness)	[52]
*Arthrospira platensis*	11.8% (starter, for 21 days)/ 9.70% (finisher, for 14 days)	Broilers-1-day old-5 weeks	Microalga improved meat quality: increased pH, water-holding capacity and less off-flavors; Increased the intensity of color in the breast meat	[53]
*Arthrospira platensis*	14.0 and 17.0% (starter, for 7 weeks)/ 12.0 and 12.8% (finisher, for 5 weeks)	Broilers-4–12 weeks old	Spirulina carotenoids are incorporated into broiler tissue; meat quality traits were not negatively affected	[51]
*Arthrospira platensis*	15.0%	Broilers-21 days old-2 weeks	Microalga increased yellowness, total carotenoids and SFA and decreased *n-*3 PUFA and α-tocopherol in breast and thigh muscles	[22]
*Chlorella vulgaris*	0.1 and 0.2%	Male Pekin ducks-1-day old-6 weeks	Microalga increased the lightness and yellowness in the leg meat and the yellowness, pH, shear force and water-hold capacity in the breast meat	[55]
*Chlorella vulgaris*	10.0%	Broilers-21 days old-2 weeks	Microalga increased tenderness, yellowness and total carotenoids in breast and thigh meat	[23]
*Schizochytrium* sp.	0.1 and 0.2%	Broilers-1-day old-5 weeks	Microalga increased oleic acid, DHA and *n*-3 PUFA; decreased SFA and *n*-6/*n*-3 ratio in breast meat	[40]
*Schizochytrium* sp.	2.8 and 5.5%	Broilers-21 days old-3 weeks	Microalga increased *n-*3 PUFA and decreased flavor scores (2.8% were still considered acceptable by sensorial panelists)	[41]
*Schizochytrium* sp.	2.00%	Broilers-21 days old-3 weeks	DHA-rich microalgae along with methionine reduced the incidence of breast muscle striping and myopathy and enriched meat with *n*-3 FA	[56]
*Schizochytrium* sp.	0.500 and 2.00%	Broilers-11 days old-4 weeks	Microalga increased *n*-3 FA, the susceptibility to oxidation in breast meat and off-flavors in thigh meat	[42]
*Schizochytrium* sp.	1.00 and 2.00%	Broilers-1-days old-5 weeks	Microalga increased *n*-3 FA in breast and thigh meat	[57]
*Schizochytrium* sp.	0.500, 2.50 and 5.00%	Broilers-1-day old-6 weeks	Microalga increased *n*-3 FA content in broiler meat	[43]
*Schizochytrium* sp.	3.70 and 7.40%	Broilers-21 days old-2 weeks	Microalga increased SFA, *n*-3 FA, LC-PUFA, EPA, DHA and TBARS; Decreased total *n*-6 FA, vitamin E, flavor and overall acceptability	[44]
*Schizochytrium* sp.	7.40%	Broilers-21 days old-2 weeks	Microalga increased the SFA, *n*-3 FA, LC-PUFA, EPA, DHA and TBARS; decreased the MUFA, total *n*-6 FA, PUFA/SFA ratio, *n*-6/*n*-3 ratio, vitamin E, flavor and overall acceptability	[45]
*Crypthecodinium cohnii*	5.00%	Muscovy ducks-50 or 43 days old-3 weeks	Microalga increased the DHA content in breast meat	[59]

## Data Availability

The data presented in this study are available on request from the corresponding author.
